# Prevalence and factors associated with warts in primary school children in Tema District, Sohag Governorate, Egypt

**DOI:** 10.1186/s42506-018-0007-0

**Published:** 2019-01-29

**Authors:** Nagwa Essa, Medhat A. Saleh, Rasha M. Mostafa, Emad A. Taha, Taghreed A. Ismail

**Affiliations:** 1 Department of Dermatology, Venereology and Andrology, Faculty of Medicine, Assiut University, Asyut, Egypt; 2 Department of Public Health and Community Medicine, Faculty of Medicine, Assuit University, Asyut, Egypt; 3 Dermatology Clinic, Tema General Hospital, Tema District, Sohag Governorate Egypt

**Keywords:** Warts, Prevalence, Risk factors, Primary school children

## Abstract

**Background:**

Warts are one of the most common, persistent, and frustrating cutaneous problems encountered in dermatology clinical practice especially in younger generations.

**Objective:**

To determine the prevalence of warts in primary school children in Tema District, Sohag, Egypt, and to determine possible factors associated with transmission.

**Participants and methods:**

A school-based cross-sectional (prevalence) study was carried out during the academic year 2015–2016 in six primary schools in Tema District, Sohag Governorate, Egypt. A total of 1045 students were examined. Data was collected using a self-administered semi-structured questionnaire which was taken home by the student to be completed by his/her caregiver.

**Results:**

Among 1045 examined school students, 108 students were diagnosed as having warts with a prevalence rate of 10.3%. Common wart was the most common type among students (49.0%) followed by plantar and plane warts (24.1%, each) while genital wart was the least one (2.8%). There was no significant sex or age difference. The prevalence of warts was significantly higher among students from public schools, rural areas, and big families; students with lower paternal education level; and students who were sharing shoes, walking barefoot, having contact with house pets, or exposed to water channels.

**Conclusion:**

Warts, especially the common variant, are highly prevalent in primary school children. The significant factors associated with the development of warts in these children were big family size and sharing shoes. Other significant associated factors included living in rural areas, attending public schools, illiterate parents, fathers with manual work, and swimming in water canals.

## Introduction

Warts are a common and distressing cutaneous problem in the general population, especially among children [1]. Despite variable reported prevalence rates in primary school children (2–20%), warts in that age seem to have an even higher prevalence than in adults [2]. Prevalence rates vary among different studies due to the variations in risk factors, sociodemographic factors, and availability of medical facilities among studied populations with the fact that only a proportion of infected children are detected when they seek medical advice or treatment for warts [1]. Although cutaneous warts usually have a benign natural history that is mostly self-limited, they may cause significant physical and psychological inconvenience [3]. The most popular cutaneous wart phenotypes in children include common warts, plantar warts, and plane or flat warts [4].

Children represent a large and important fraction of the population especially in developing countries like Egypt [2]. Determination of the prevalence of a health problem in a given population and understanding the risk factors for its transmission enable the health authorities to make strategies to solve it [5]. Epidemiological data on the prevalence of cutaneous warts in Egypt especially in children are limited, and available studies are conducted in selected patient groups mostly from dermatology clinics or small urban areas in Lower Egypt cities [2]. Few community-based studies assessing the prevalence and risk factors of warts are available; one of them reported a prevalence rate of warts about 7.4% among primary school children in Bab El-Shaareya region, Cairo city [5], and another one was conducted in rural Lower Egypt with a reported prevalence rate of 2.3% in the hands only [2]. A study in Mansoura was limited to children with disabilities [6].

Risk factors of HPV infection include immunosuppression, close contact with affected people, and activities such as nail biting and walking barefoot [7]. Moreover, Kasim et al. [2] demonstrated a significant increase in the risk of warts among rural Egyptian children who were swimming in Nile channels and who gave a history of child labor. The aims of the present study are to determine the prevalence of warts among primary school children and to determine the possible factors associated with the development of warts in Tema District, Sohag Governorate, Upper Egypt.

### Participants and methods

#### Study design and setting

This was a school-based cross-sectional study conducted in 2015–2016 involving six primary schools chosen randomly to represent different sectors (rural and urban, boys and girls, public and private education, age groups with class grades 1–6) of primary school children population in Tema District, a district of Sohag Governorate. Sohag is located in the middle of “Upper Egypt,” and it includes 11 districts. Tema is the first district from the north, and it includes 36 villages.

We calculated the required sample size using the EPI info using the following data: the study population was about 15,000 (the number of the primary school students in Tema District), the estimated prevalence of warts in Egypt was 7.4% [5], and a confidence level was set at 99.0%. The calculated sample was 180 students. In the present study, a total of 1045 students were examined to allow better stratification of many independent variables (age group, residence, gender, school type, and family members) and determination of many possible associated factors. A stratified random sampling technique that included governmental and English governmental schools (Tajreeby) and private schools was applied. The present study was carried out in six primary schools (five governmental and one private schools). The total sample was distributed among governmental and private education schools proportionate to the number of students in each type of education and in the randomly chosen schools proportionate to the numbers of the students in each school. Among a total of 1045 student, 196 were from a private school, 174 were from a governmental language school, and the remaining 675 were belonging to governmental schools.

The sample unit was classes that were also randomly chosen. The researchers examined one or more class in each grade according to the sample size calculated for each academic grade and the number of students in each class. The selected classes were examined almost totally excluding those who refused to participate in the study.

Data was collected using a self-administered semi-structured questionnaire which was taken home by the student to be completed by his/her caregiver. The questionnaire included personal, sociodemographic, and environmental data in addition to the data about risk factors exposure, similar infection among family members. The examination was done to all students to diagnose warts, its type, and site. Physical examination of the students was carried out by the researcher in the school clinics to keep the privacy of the students.

A pilot study was carried out on 50 primary school students to test the questionnaire for any required modifications. Minor modifications and rephrasing were done. The pilot sample was not included in the study sample.

#### Statistical analysis

Data was managed using SPSS version 20 (SPSS, Inc., Chicago, IL, USA). Quantitative variables were expressed as mean ± SD, and qualitative variables were presented as number and percentage. The chi-square test was used to compare frequencies. A binary logistic regression model was used to determine the odds ratio for each associated factor. *P* value was considered statistically significant when ≤ 0.05.

## Results

This study included 1045 children with age range from 6 to 12 years and a mean age of 9.32 ± 1.85 years. About 55% were boys and 45% were girls, and 48% of students were from rural areas and 52% were from urban areas. About 81% of students were from public schools versus 19% from a private school. Of the 1045 examined children, 108 students were diagnosed as having warts giving a prevalence rate of 10.3%. There was no significant difference in wart prevalence according to sex and age, while the prevalence was significantly higher in students from rural areas, public schools, and big families (7 or more members per family) (14.0%, 11.4%, and 23.1%, respectively) (Table 1).

**Table 1:** Relation between wart infection and sociodemographic characteristics of primary school students, academic year 2015–2016, Tema District, Sohag, Egypt

	Total, *N* = 1045		With wart, *N* = 108		*P* value
	No.	%	No.	%	
Age (years)					0.07
6 to < 8	374	35.8	32	8.6	
8 to 10	342	32.7	46	13.5	
> 10	329	31.5	30	9.1	
Sex					0.14
Boys	579	55.4	67	11.6	
Girls	466	44.6	41	8.8	
Residence					< *0.001*
Rural	501	48.0	70	14.0	
Urban	544	52.0	38	7.0	
School type					*0.02*
Public	849	81.2	97	11.4	
Private	196	18.8	11	5.6	
Family members					< *0.001*
3–4	374	358	8	4.8	
5–6	342	32.7	49	7.4	
7 or more	329	31.5	51	23.1	

**Fig. 1:**
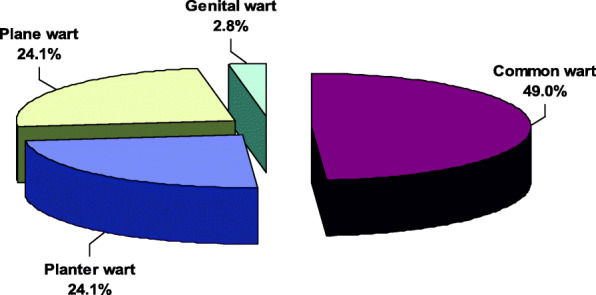
Type of warts among primary school students, Tema District, Sohag, Egypt

**Fig. 2:**
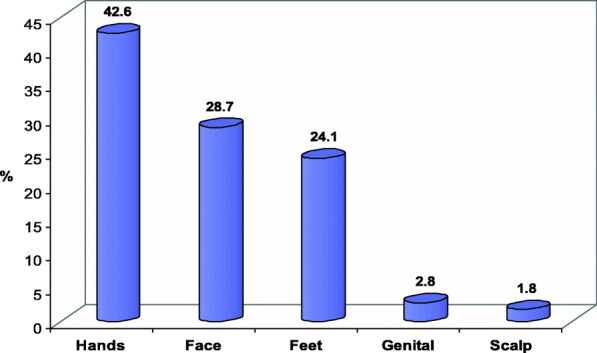
Site of warts among primary school students, Tema District, Sohag, Egypt

Common wart was the most common type of warts among students (49.0%) followed by plantar and plane warts (24.1% each) while genital wart was the least frequent (2.8%) (Fig. 1). The hand was the most commonly affected (42.6%) followed by the face (28.7%) and feet (24.1%). The genital area and scalp were the lowest affected sites (2.8% and 1.9%, respectively) (Fig. 2).

**Fig. 3:**
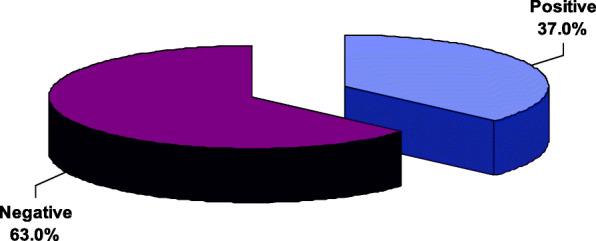
History of positive family contact among affected students, Tema District, Sohag, Egypt

Paternal education and job showed a significant impact on wart infection (wart infection was significantly higher among students whose fathers were illiterate, farmers, and unskilled workers as shown in Table 2). Certain lifestyle factors were associated with a significant increase in wart prevalence such as sharing shoes or walking barefoot, exposure to water canals, and presence of pets in the house (22.7%, 13.0%, 23.2%, and 16.3%, respectively) (Table 3). History of positive family contact was reported among 37.0% of the affected students (Fig. 3).

**Table 2:** Relation between wart infection of primary school students and parent’s education and job, academic year 2015–2016, Tema District, Sohag, Egypt

	Total		With wart		*P* value
	No.	%	No.	%	
Father’s education	(1013)^≠≠^		(107)^≠^		
University education	380	37.5	26	6.8	*< 0.001*
Secondary/equivalent	445	43.9	45	10.1	
Illiterate	188	18.6	36	19.1	
Father’s job	(1013)^≠≠^		(107)^≠^		*< 0.001*
Employee	375	37.0	23	6.1	
Farmer	139	13.7	27	19.4	
Skilled worker	191	18.9	20	10.5	
Free business	253	25.0	26	10.3	
Unskilled worker**/**unemployed	55	5.4	11	20.0	
Mother’s education	(1043)^≠≠≠^		(108)		*< 0.001*
University	389	37.3	22	5.7	
Secondary/equivalent	494	47.4	56	11.3	
Illiterate	160	15.3	30	18.8	
Mother’s job	(1043)^≠≠≠^		(108)		*< 0.001*
Housewife	619	59.3	90	14.5	
Working	424	40.7	18	4.2	

^≠^One dead father

^≠≠^Thirty-one dead fathers

^≠≠≠^Two dead mothers

**Table 3:** Relation between wart infection and some lifestyle factors among primary school students, academic year 2015–2016, Tema District, Sohag, Egypt

	Total (1045)		With wart (108)		*P* value
	No.	%	No.	%	
Wearing shoes habit					*< 0.001*
Always wearing own shoes	730	69.9	47	6.4	
Sometimes barefoot	108	10.3	14	13.0	
Sharing shoes	207	19.8	47	22.7	
Exposure to water canal					*< 0.001*
Yes	272	26.0	63	23.2	
No	773	74.0	45	5.8	
Exposure to house pets					*< 0.001*
Yes	406	38.9	66	16.3	
No	639	61.1	42	6.6

The binary logistic regression model shows that the odds ratio of wart infection is significantly higher in students who live in big families (OR = 3.5) and those who are sharing shoes with other family members (OR = 2.8) (Table 4).

**Table 4:** Logistic regression of factors associated with wart infection among primary school students, academic year 2015–2016, Tema District, Sohag, Egypt

	*P* value	OR	95% confidence interval	
			Lower	Upper
Rural area	0.755	1.089	0.637	1.863
Public school	0.881	1.061	0.489	2.301
Father education	0.187			
Secondary/equivalent	0.067	0.485	0.223	1.052
Illiterate	0.190	0.532	0.207	1.367
Mother education	0.892			
Secondary/equivalent	0.690	1.180	0.524	2.658
Illiterate	0.635	1.270	0.473	3.413
No. of family members	< *0.001*			
5–6	0.452	1.354	0.615	2.983
7 or more	*0.004*	*3.513*	1.506	8.194
House pets	0.098	1.516	0.926	2.484
Share shoes	< *0.001*	*2.844*	1.795	4.506

## Discussion

Warts are one of the most chronic and frustrating skin and mucosal conditions encountered in dermatology clinics [8]. Despite variable reported prevalence rate in primary school children, warts in this age seem to have an even higher prevalence than in adults. In our study, we reported a total prevalence of warts, 10.3%. This agrees with the finding reported in Medinah and Jeddah regions, Saudi Arabia [9]. On the other hand, a much lower prevalence rate (4.5%) was reported in Al Hassa rural area, Saudi Arabia [10], whereas a higher prevalence (13.1%) was reported in Kuwait [11].

Some studies from other areas in the world reported variable results. The prevalence of warts in primary school children in Romania and Taiwan was 6.9%, 2.4%, and 2.8% , respectively [12–14]. The highest reported prevalence was 33% among Dutch primary school students [1].

The difference in the prevalence rate of warts between different studies may reflect the difference in sociodemographic patterns and distribution of risk factors among studied children in addition to differences in the inclusion criteria of the target population.

In the present study, neither age nor sex showed a statistically significant difference regarding wart prevalence. On the other hand, some other studies reported a higher prevalence in older age groups from 8 to 12 years [15, 16] or with male sex [9] as males are more exposed to outdoor activities that may carry a risk for infection such as exposure to water channels, manure, and animals.

Common wart was the most prevalent type in our study. This is in line with the majority of published studies in school children [5, 15, 17] with some exceptions that reported higher percent of affection with plantar warts than common warts [18].

Fortunately, genital warts had the lowest percent in our study (2.8%) despite it was reported to be the second prevalent type in children after common wart in a study in Kuwait [11] where the presence of foreign babysitters and servants from different nationalities is more common [15] than that in Egypt.

Genital warts are of special importance since children with genital warts will often raise the suspicion of child sexual abuse [19]. However, several studies indicate that the origin of pediatric anogenital human papilloma viral infections remains often untraced (innocent warts), with no indication of sexual abuse [4].

The hand was the most commonly affected site in our study. This agrees with many other studies [15, 17, 20]. Hands are the most common site that had a high likelihood to contact a contaminated environmental surface during play or work besides the natural tendency for children to pick or scratch at existing warts.

The impact of socioeconomic status on the prevalence of warts is evident in our study as the prevalence was higher in children from rural areas, public schools, and big families. Similar results were also reported in many other studies [9, 10, 18, 21]. Factors like overcrowding, lower hygiene with sharing of personal fomites, and reluctance to seek medical advice are more common in children from rural areas, public schools, and big families which reflect lower socioeconomic level. Also, the level of paternal and maternal education and work status had an impact on wart prevalence as high education, skilled father work, and working mothers were associated with a low prevalence of warts. This was concluded in many other studies [2, 9, 10, 21].

Education and socioeconomic state of the individuals certainly affect health awareness and standards of hygiene within the family; educated parents will seek medical advice if their son/daughter has wart [22, 23]. Although this attitude is less likely to decrease wart incidence, it may decrease wart prevalence through shortening the disease duration [22].

Exposure to water channels, walking barefoot on soil, and sharing shoes with other family members were significant associated factors for wart infection in our study. These factors are more common in rural areas and big families. The results of other studies about these risk factors were contradictory [2, 15, 18].

However, studies on environmental risk factors for warts are contradictory and all have a cross-sectional design where the causal influences of the risk factors could not be exactly determined.

Definite positive family contact could be detected among 37% of the affected students in our study. Unfortunately, we could not get definite information about the presence or absence of positive family contacts in non-affected children, so we could not obtain an odds ratio for that risk factor. Two studies reported that the presence of positive family contacts is the major positive predictor for wart prevalence in school children regardless of other environmental factors such as use of public swimming pools, practice sports barefoot, and use of public showers [1, 18].

By regression analysis of different associated factors, big family size was a significant associated factor for wart infection in our study. The same finding was also concluded in many other studies [2, 9, 10, 18, 21]. Another significant associated factor was sharing shoes with other family members (22.7%) which is also more likely to occur in large families and low socioeconomic level.

### Limitations of the study

Data on the risk factors were collected by recall which may be subjected to some bias. The results represent the prevalence in Tema District and cannot be generalized to Sohag Governorate as a whole.

## Conclusion

The prevalence of warts was 10.3% among primary school children in Tema, a mixed rural-urban area of Upper Egypt. Common wart was the most common type, and the hand was the most affected site. Significant predictors were big family size and sharing shoes with other family members. Other significant associated factors included living in rural areas, belonging to public schools, illiterate parents and fathers with unskilled work, contact with animal pets, and swimming in water canals.

## Recommendations

Health education about the simple preventive measures and personal hygiene is important to decrease the prevalence of infectious skin diseases such as warts among children who can be easily approached in schools through curricula and activities.

## References

[1] Bruggink SC, Eekhof JAH, Egberts PF, van Blijswijk SCE, Assendelft WJJ, Gussekloo J (2013). Natural course of cutaneous warts among primary schoolchildren: a prospective cohort study. Ann Fam Med.

[2] Kasim K, Amer S, Mosaad M, Abdel-Wahed A, Allam H. Some epidemiologic aspects of common warts in rural primary school children. ISRN Epidemiol. 2013;2013:1–6. Article ID 283591. 10.5402/2013/283591

[3] Bruggink SC, de Koning MN, Gussekloo J, Egberts PF, TerSchegget J, Feltkamp MC (2012). Cutaneous wart-associated HPV types: prevalence and relation with patient characteristics. J Clin Virol.

[4] Syrjänen S (2010). Current concepts on human papillomavirus infections in children. APMIS.

[5] Makhlouf NN (2007). The prevalence of dermatological diseases among school children in Bab El-Shaareya region, Cairo city thesis [M.S. of dermatology and venereology].

[6] Fathy H, El-Mongy S, Baker NI, Abdel-Azim Z, El-Gilany A (2004). Prevalence of skin diseases among students with disabilities in Mansoura. Egypt East Mediterr Health J.

[7] Lynch MD, Cliffe J, Morris-Jones R (2014). Management of cutaneous viral warts. Brit Med J.

[8] Han TY, Lee JH, Lee CK, Ahn JY, Seo SJ, Hong CK (2009). Long-pulsed Nd:YAG laser treatment of warts: report on a series of 369 cases. J Korean Med Sci.

[9] Allayali AZ, Fallatah K, Alorfi S, Mogharbel B (2017). Prevalence and risk factors of *Verruca vulgaris* among primary school children in Medinah and Jeddah, Saudi Arabia. J Clin Exp Dermatol Res.

[10] Amin TT, Ali A, Kaliyadan F (2011). Skin disorders among male primary school children in Al Hassa, Saudi Arabia: prevalence and socio-demographic correlates--a comparison of urban and rural populations. Rural Remote Health.

[11] Nanda A, Al-Hasawi F, Alsaleh QA (1999). A prospective survey of pediatric dermatology clinic patients in Kuwait: an analysis of 10,000 cases. Pediatr Dermatol.

[12] Popescu R, Popescu CM, Williams HC, Forsea D (1999). The prevalence of skin conditions in Romanian school children. Br J Dermatol.

[13] Wu YH, Su HY, Hsieh YJ (2000). Survey of infectious skin diseases and skin infestations among primary school students of Taitung County, eastern Taiwan. J Formos Med Assoc.

[14] Yang Y-C, Cheng Y-W, Lai C-S, Chen W (2007). Prevalence of childhood acne, ephelides, warts, atopic dermatitis, psoriasis, alopecia areata and keloid in Kaohsiung County, Taiwan: a community-based clinical survey. J Eur Acad Dermatology Venereol.

[15] Al-Mutairi N, AlKhalaf M (2012). Mucocutaneous warts in children: clinical presentations, risk factors, and response to treatment. Acta dermatovenerologica Alpina, Pannonica, Adriat.

[16] Ezz El-Dawela R, Fatehy AN, Elmoneim AAA (2012). Prevalence of skin diseases among school children. J Egypt Womenʼs Dermatologic Soc.

[17] Ghadgepatil SS, Gupta S, Sharma YK (2016). Clinicoepidemiological study of different types of warts. Dermatol Res Pract.

[18] van Haalen FM, Bruggink SC, Gussekloo J, Assendelft WJJ, Eekhof JAH (2009). Warts in primary school children: prevalence and relation with environmental factors. Br J Dermatol.

[19] Mammas IN, Sourvinos G, Spandidos DA (2009). Human papilloma virus (HPV) infection in children and adolescents. Eur J Pediatr.

[20] Bacelieri R, Johnson SM (2005). Cutaneous warts: an evidence-based approach to therapy. Am Fam Physician.

[21] Chen G-Y, Cheng Y-W, Wang C-Y, Hsu T-J, Ming-Long Hsu M, Yang P-T (2008). Prevalence of skin diseases among school children in Magong, Penghu, Taiwan: a community-based clinical survey. J Formos Med Assoc.

[22] Cochrane SH, Leslie J, O’Hara DJ (1982). Parental education and child health: intracountry evidence. Health Policy Educ.

[23] Chou S-Y, Liu J-T, Grossman M, Joyce T (2010). Parental education and child health: evidence from a natural experiment in Taiwan. Am Econ J Appl Econ.

